# Biological Sex, Estradiol and Striatal Medium Spiny Neuron Physiology: A Mini-Review

**DOI:** 10.3389/fncel.2018.00492

**Published:** 2018-12-12

**Authors:** Amanda A. Krentzel, John Meitzen

**Affiliations:** ^1^Department of Biological Sciences, North Carolina State University, Raleigh, NC, United States; ^2^W. M. Keck Center for Behavioral Biology, North Carolina State University, Raleigh, NC, United States; ^3^Center for Human Health and the Environment, North Carolina State University, Raleigh, NC, United States; ^4^Comparative Medicine Institute, North Carolina State University, Raleigh, NC, United States

**Keywords:** female, estradiol, estrous cycle, spiny projection neurons, caudate-putamen, dorsal striatum, nucleus accumbens, aromatase

## Abstract

The caudate-putamen, nucleus accumbens core and shell are important striatal brain regions for premotor, limbic, habit formation, reward, and other critical cognitive functions. Striatal-relevant behaviors such as anxiety, motor coordination, locomotion, and sensitivity to reward, all change with fluctuations of the menstrual cycle in humans and the estrous cycle in rodents. These fluctuations implicate sex steroid hormones, such as 17β-estradiol, as potent neuromodulatory signals for striatal neuron activity. The medium spiny neuron (MSN), the primary neuron subtype of the striatal regions, expresses membrane estrogen receptors and exhibits sex differences both in intrinsic and synaptic electrophysiological properties. In this mini-review, we first describe sex differences in the electrophysiological properties of the MSNs in prepubertal rats. We then discuss specific examples of how the human menstrual and rat estrous cycles induce differences in striatal-relevant behaviors and neural substrate, including how female rat MSN electrophysiology is influenced by the estrous cycle. We then conclude the mini-review by discussing avenues for future investigation, including possible roles of striatal-localized membrane estrogen receptors and estradiol.

## Introduction

**Figure 1:**
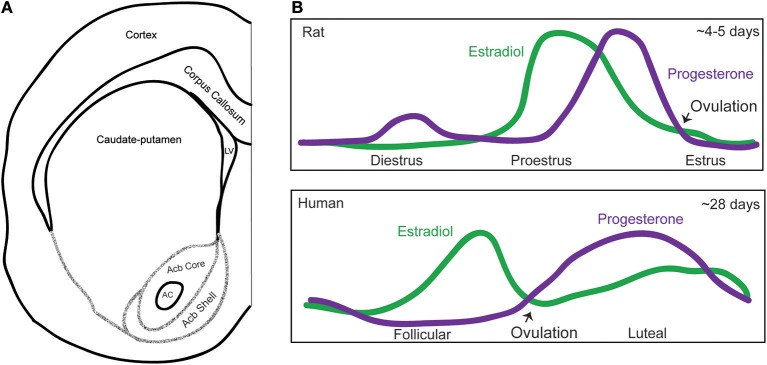
Map of the striatal subregions and female hormone cycling. **(A)** Schematic of a coronal section of one hemisphere of the rat brain depicting the striatal subregions, including the caudate-putamen, nucleus accumbens core, and shell (Interaural ~10.92–10.80 mm, Bregma ~1.92–1.80 mm). Acronyms: AC, anterior commissure; Acb, nucleus accumbens; LV, lateral ventricle. The extensive afferent and efferent circuitry of the striatal subregions is not depicted in this schematic, and we refer the reader to the following articles for a review of this topic (Russo and Nestler, 2013; Scofield et al., 2016) **(B)** Graphical depictions of the adult female rat estrous and human menstrual cycle. Purple line indicates progesterone levels and the green line estradiol levels. Over a span of about 4–5 days, rats exhibit a diestrus, proestrus, and estrus phase. There is also a metestrus phase between estrus and diestrus (not pictured). In rats, estradiol levels peak the morning of proestrus, as progesterone levels are rising, and behavioral estrus begins roughly when progesterone levels peak. The human cycle lasts about 28 days, and exhibits a follicular and luteal phase. In humans, estradiol peaks during the follicular phase, and progesterone peaks during the luteal phase.

Sex differences in brain structure and function have been described at all levels of biological analysis, from differences in neuronal gene expression to the output of the nervous system, behavior (McCarthy, 2010; Forger, 2016; Arnold, 2017; Grabowska, 2017). Sex is a compelling biological variable that must be considered from single neuron analysis all the way to clinical trials. The striatal regions, including the caudate-putamen and nucleus accumbens core and shell (Figure 1A), are sensitive to biological sex and sex steroid hormone fluctuations and signaling in both animals and humans. Although striatal sex and hormone-specific differences have long been documented, the mechanisms by which hormones and sex influence caudate-putamen and accumbens physiology remain active research areas. In this mini-review, we first describe the known sex differences in the physiology of the output neuron of the striatal brain regions, the medium spiny neuron (MSN), in prepubertal rats. We then broaden the discussion to address aspects of how the menstrual cycle in adult female humans and estrous cycle in adult female rats influences striatal-relevant behaviors, and feature select studies providing mechanistic insight. This includes recent data demonstrating that the estrous cycle modulates MSN physiology. We then end the mini-review by presenting two challenge hypotheses for future investigation, namely, the possible roles of striatal-localized membrane estrogen receptors and neuroestrogen production.

## Caudate-Putamen and Nucleus Accumbens Core MSNs Exhibit Sex Differences Before Puberty

MSNs (or alternatively, spiny projection neurons) consist of ~95% of striatal neurons (Kemp and Powell, 1971; Graveland and DiFiglia, 1985; Gerfen and Surmeier, 2011) and are the major efferent projection neurons. MSNs do not exhibit gross sex differences in soma size or neuron density (Meitzen et al., 2011), and the overall volume of the striatal brain regions does not robustly differ between males and females (Wong et al., 2016). MSNs do exhibit functional electrophysiological properties that differ by striatal subregion and developmental period (Table 1). Before puberty, sex differences are present in both intrinsic and synaptic properties of MSNs that is specific to striatal region in rats. Here we define intrinsic properties are those being related to single action potential properties such as threshold, multiple action potential properties such as action potential firing rate as evoked by excitatory current injection, and passive membrane properties such as input resistance. All of these properties are unified in that they help determine how a neuron responds to synaptic input, in other words, the input-output process of the individual neuron. Regarding synaptic properties, here we focus on properties that have been directly investigated in MSN with regards to sex, such as miniature excitatory postsynaptic currents (mEPSC), which provides insight into the strength, number, and sensitivity of glutamatergic synapse. In rat caudate-putamen, MSN excitability is increased in females compared to males, as indicated by an increased evoked action potential to excitatory current injection slope, hyperpolarized threshold, and decreased after hyperpolarization magnitude in females compared to males. There are no differences in mEPSC properties, including frequency, amplitude, and decay (Dorris et al., 2015). Conversely, in the nucleus accumbens core, mEPSC frequency is increased in prepubertal females compared to males and this sex difference exist both pre-puberty and in adults (Cao et al., 2016). This sex difference is organized during the postnatal critical window (P0–P1) and in females can be eliminated by postnatal 17β-estradiol (estradiol) or testosterone exposure (Cao et al., 2016). Estradiol is a type of estrogen, which binds to estrogen receptors. Testosterone can either bind to androgen receptors or be metabolized via the enzyme aromatase into estradiol to in turn act on estrogen receptors. Prepubertal recordings from nucleus accumbens shell did not show any sex differences in MSN electrical properties (Willett et al., 2016), however environmental influences such as stress engender sex differences in synapse markers in adult rodents (Brancato et al., 2017). Together, these studies illustrate heterogeneity of sex-specific mechanisms across the subregions of the striatum (Cao et al., 2018b). Interestingly, sex differences in MSN properties detected in prepubertal rat are different than those detected in prepubertal mouse nucleus accumbens core (Cao et al., 2018a), indicating that sex differences in the development of MSN electrophysiological properties can be species-specific or perhaps mouse strain-dependent. It is also unknown how sex differences and sex steroid sensitivity present across MSN subtypes. This question is an important avenue for future investigations, as differential sensitivity to biological sex across MSN subtypes may have important functional consequences.

**Table 1:** Sex differences of electrophysiological properties of medium spiny neurons across striatal subregions in rats.

**Electrophysiological property**	**Developmental stage**	**Caudate-putamen**	**Nucleus accumbens core**	**Nucleus accumbens shell**
Intrinsic excitability	Prepubertal	F > M	F = M	F = M
	Adult	?^d^	Cycle determines sex difference^a^	?
Excitatory synaptic input	Prepubertal	F = M	F > M^b^	F = M
	Adult	?	Cycle determines sex difference*^a^*	?^c^

*Gray fill indicates sex and/or cycle dependent differences. Inequality signs indicate relative differences between sexes. “?” indicates complex or no evidence*.

a *Estrous cycle stage determined directionality of sex difference and difference between female estrous stages. Gonadectomy eliminates sex differences*.

b *This sex difference has been shown to be organized by estradiol during masculinization window*.

c *Examination of synapse properties shows divergent evidence of sex differences in non-stressed animals, but an electrophysiological approach in adult animals has not yet been done to our knowledge (as reviewed by Cao et al., 2018b). The adult nucleus accumbens shell exhibits variable sex differences, likely indicating interactions with other environmental influences such as stress (i.e., Brancato et al., 2017)*.

d *In adult caudate-putamen, estrous-cycle induced differences in select rat medium spiny neuron action potential generation rates have been reported in vivo, but the underlying cellular electrophysiological mechanisms are not yet documented*.

## The Menstrual and Estrous Cycles Influence Striatal-Related Behaviors and Disorders in Adult Females

In adult female humans, the cyclical fluctuation of estradiol, progesterone, and other hormones is called the menstrual cycle and is ~28 days long. Plasma estradiol levels peak during the follicular phase, while progesterone levels peak during the luteal phase (Sherman and Korenman, 1975). In adult female rats and mice, this cycle is called the estrous cycle and likewise features repeated hormone changes, but across a ~4–5 day period (Cora et al., 2015). In rats, plasma estradiol levels rapidly peak during proestrus, after which progesterone levels peak, leading to ovulation and a resulting estrus phase. The diestrus phase, during which hormone levels are generally low, follows the estrus phase (Figure 1B).

Regarding behaviors associated with the striatal regions, changes in motor coordination and severity of Parkinson's symptoms, which are controlled by the caudate-putamen, have been associated with the menstrual cycle. The luteal phase, when estradiol and progesterone are high, is associated with more coordination, manual skills, and less L-DOPA-induced dyskinesia (Quinn and Marsden, 1986; Hampson and Kimura, 1988; Hampson, 1990). These findings in menstrual cycle-related behavioral changes generalize to other movement disorders with worsening of symptoms occurring just before and during menses when estradiol and progesterone are lowest (Castrioto et al., 2010). Additionally, changes in anxiety-related behaviors and anxiety-related symptoms which are controlled, in part, by the nucleus accumbens, also occur across the menstrual cycle (Nillni et al., 2011). In general, the extent of documented changes in motor skills and cognitive functions across the human menstrual cycle differs across population characteristics and sampled task-type (Souza et al., 2012).

## Dopamine and Estradiol Are Part of the Mechanism Underlying Female Cycle-dependent Differences

Animal studies have provided more controlled designs and techniques to understand the mechanisms underlying these sex differences. It has long been documented that the dopamine and estrogen systems interact to influence striatal function (Yoest et al., 2018b). Here we highlight some select pieces of evidence. In female monkeys, during the luteal phase, D2 receptor availability is increased in the caudate-putamen and nucleus accumbens (Czoty et al., 2009) suggesting that gonadal hormones may influence dopamine (DA) transmission and sensitivity which can promote movement coordination. In rats, females during proestrus and estrus (comparable to luteal phase in humans and monkeys) have higher extracellular DA concentrations than diestrus and ovariectomized females (Xiao and Becker, 1994). Estrous cycle-dependent changes in dopamine signaling have also been observed in mice (Calipari et al., 2017). This may be a mechanism that contributes to changes in locomotion (Becker et al., 1987) and anxiety (Marcondes et al., 2001; Sayin et al., 2014) across estrous cycle in rodents. Gonad-intact and castrated males do not differ, indicating that gonadal hormone influences on striatal release of dopamine are sex-specific (Xiao and Becker, 1994). Estradiol has been proposed as a major hormone to facilitate sex differences. Specific to the caudate-putamen, estradiol promotes motor coordination (Becker et al., 1987; Schultz et al., 2009) and its enhancement of dopamine action is specific to females (Becker, 1990; Xiao and Becker, 1994; Yoest et al., 2014, 2018a). The role of dopamine in regulating MSN electrical properties suggests that MSN properties would likewise differ between males, females, and across the adult female hormone cycle (Nicola et al., 2000).

## Cyclical Female Hormone Fluctuations Induce Sex Differences in Adult MSN Electrical Properties

Intrinsic and synaptic electrophysiological properties of MSNs of the caudate-putamen and nucleus accumbens core change with the estrous cycle (Arnauld et al., 1981; Tansey et al., 1983; Proaño et al., 2018). In the caudate-putamen, classic experiments first demonstrated that spontaneous action potential firing rates recorded *in vivo* increased in ovariectomized female rats exogenously exposed to estradiol compared to vehicle-exposed females and males (Arnauld et al., 1981). Later on, using *in vivo* extracellular recording, it was found that nigrostriatal MSNs increased spontaneous action potential generation in female rats during the phases of the estrous cycle associated with high levels of estradiol, or in ovariectomized females exposed to exogenous estradiol compared to animals with low levels of estradiol (Tansey et al., 1983). Other MSN subtypes and striatal interneurons were not tested in this study. The exact electrophysiological, endocrine, and molecular mechanisms driving these changes in electrical activity in the caudate-putamen remain to be elucidated, although this is an area of active research. More detailed data is available for MSNs in the adult female rat nucleus accumbens. In the nucleus accumbens core, during diestrus, when both progesterone and estradiol are low, MSN excitatory synaptic input properties decrease in magnitude while intrinsic excitability increases (Proaño et al., 2018). Specifically, mEPSC frequency and amplitude are decreased compared to other estrous cycle phases, while properties such as action potential rheobase, action potential threshold, input resistance, and resting membrane potential change to increase cellular excitability. Conversely, during proestrus and estrus, which are when estradiol and progesterone increase, and females are sexually receptive, excitatory synaptic input increases and intrinsic excitability decreases. mEPSC frequency and amplitude are increased compared to other estrous cycle phases, aligning with previous work examining excitatory synapse anatomy in females in these estrous cycle phases solely compared to males (Forlano and Woolley, 2010; Wissman et al., 2012). In contrast, cellular properties such as action potential rheobase, action potential threshold, input resistance, and resting membrane potential change to decrease cellular excitability. When analyzing these properties in gonadectomized males and females, all sex differences disappear (Proaño et al., 2018). This study indicates that adult female hormone cycles are necessary to induce sex differences in adult MSN properties, including excitatory synapse function. Changes in excitatory synaptic properties are consistent with previous anatomical studies in adult rats (Forlano and Woolley, 2010; Staffend et al., 2011; Wissman et al., 2011, 2012; Martinez et al., 2016; Peterson et al., 2016). Whether these properties differ by MSN subtype is still unknown. Given that accumbens core MSNs exhibit divergent sex differences across development, sexual differentiation of MSNs likely occur across multiple developmental periods. Puberty may be one such period (Ernst et al., 2006; Kuhn et al., 2010; Manitt et al., 2011; Matthews et al., 2013; Staffend et al., 2014; Kopec et al., 2018).

## Challenge Hypothesis #1: How Do Membrane Estrogen Receptors Influence Striatal Neuron Physiology?

Although there is ample evidence that estradiol is an important and sex-specific hormonal regulator of striatal behavior, dopamine systems, and MSN function, the exact mechanisms by which estradiol exerts its actions requires further research. An increasing body of work strongly implicates membrane estrogen receptor action. Adult female rats exclusively express membrane estrogen receptors (GPER1, membrane-associated ERα, and membrane-associated ERβ) in MSNs of the caudate-putamen and accumbens (Almey et al., 2012). However, to our knowledge a thorough analysis of estrogen receptors across development, MSN subtype, and species has not been accomplished and nuclear estrogen receptors may be expressed at early developmental stages. Sex-specific differences in membrane estrogen receptor facilitation of changes in neuronal activity have been reported in other brain regions (Oberlander and Woolley, 2016; Krentzel et al., 2018). Importantly, sex differences in function can exist even when receptor expression is similar between males and females (Krentzel et al., 2018), indicating that the sex-specific sensitivity and functionality of estrogen receptors are more complicated than indicated by anatomical analyses alone.

Membrane estrogen receptors are expressed both on axon terminals, MSN somas and dendritic spines (Almey et al., 2012, 2015, 2016), and there is evidence that estradiol has both pre- and post-synaptic mechanisms for altering dopaminergic signaling which promotes locomotion (Becker and Beer, 1986). Estrogen receptors associated in the membrane with metabotropic glutamate receptors have also been shown to facilitate locomotor sensitization to cocaine (Martinez et al., 2014), involved in drug addiction (Tonn Eisinger et al., 2018), and change dendritic spine morphology in the nucleus accumbens (Peterson et al., 2015). Application of estradiol increases dopamine (DA) rapidly in the accumbens and caudate-putamen (Becker, 1990; Pasqualini et al., 1996), as well as decreases GABA production (Hu et al., 2006). This suggests that estradiol may indirectly act on dopamine signaling by first releasing inhibition from GABAergic signaling, and perhaps also directly upon dopamine-producing regions. In striatal MSNs, estradiol acting through ERα, ERβ, and mGluR rapidly decreases L-type calcium currents and phosphorylates the transcription factor CREB (Mermelstein et al., 1996; Grove-Strawser et al., 2010).

One proposed model for estradiol actions on striatal networks builds upon these and other findings, positing that estradiol binds to membrane estrogen receptors on MSNs to decrease neuronal excitation, therefore leading to less GABA release and a “disinhibition” of dopaminergic signaling either through a collateral synapse upon dopamine fibers from the substantia nigra pars compacta or the VTA (Yoest et al., 2014, 2018b). Direct evidence that estradiol rapidly acts on MSNs to decrease intrinsic neuronal excitability or excitatory post synaptic currents remains unknown, although this is an active area of research. This model also predicts that MSNs synapse upon either dopaminergic fibers from the substantia nigra pars compacta, the VTA, or perhaps tyrosine-hydroxylase positive striatal interneurons. Alternatively, estradiol may potentially act on striatal interneurons, such as the cholinergic subtype, which synapses upon both dopamine terminals and MSNs (Chuhma et al., 2011). Cholinergic interneurons express membrane estrogen receptors and have been implicated in estradiol-induced shifting between hippocampal and striatal-based learning behaviors, suggesting interactions been estrogen, cholinergic, and dopamine-systems (Euvrard et al., 1979; Davis et al., 2003; Almey et al., 2012). These models are not necessarily mutually exclusive. They also do not exclude direct actions of estradiol on MSNs independent of dopaminergic signaling, perhaps instead targeting glutamatergic systems. Consistent with this speculation, glutamatergic systems have been implicated in sex differences in psychiatric diseases such as anxiety (Wickens et al., 2018).

## What Is the Relationship Between Membrane Estrogen Receptors and the Estrous Cycle?

Gonadal hormone fluctuations related to the estrous cycle correlate with changes in both caudate-putamen and accumbens dependent behaviors and with the electrical properties of MSNs. This conclusion raises questions regarding the potential relationship between the estrous cycle and the actions of rapid estradiol signaling to modulate striatal neuron activity. To date, one study has shown that after 3 days of estradiol priming to artificially mimic estradiol-high proestrus of females, locomotion and DA release is potentiated after an acute estradiol injection and amphetamine (Becker and Rudick, 1999). This work is one piece of evidence that females may exhibit cycle-dependent rapid estradiol mechanisms. Estradiol-mediated signaling in MSNs may alter depending on estrous cycle phase, though little work has tested this hypothesis, much less uncovered the mechanistic details of how this may occur. It is unknown how cycle stage changes sensitivity to estradiol, estrogen receptor expression, and synapse functionality. However, proestrus (higher estradiol and progesterone) females exhibit more and larger dendritic spines than males (Forlano and Woolley, 2010; Wissman et al., 2011). Other estrous cycle phases were not examined. This anatomical work from Woolley and colleagues is consistent with electrophysiological findings which indicate strong sex differences during the proestrus phase (Proaño et al., 2018).

## Challenge Hypothesis #2: Does Local Production of Estradiol Influence Caudate-putamen and Nucleus Accumbens Function?

Another component of rapid estradiol signaling is the dynamic production of localized estradiol. Evidence of aromatase activity and fluctuations in local estradiol content have been shown across vertebrate brains (Callard et al., 1978) especially in songbirds (Saldanha et al., 2000; Remage-Healey et al., 2008, 2012; Ikeda et al., 2017). Low levels of aromatase, the enzyme that synthesizes estradiol from testosterone, has been observed in processes and cell bodies of rat striatum (Jakab et al., 1993; Wagner and Morrell, 1996; Horvath et al., 1997) but a thorough analysis and comparison across subregions has not been performed. It is unknown how aromatase expression differs based on age, sex, cell compartment, or cell subtype, thus overly-definitive statements regarding striatal aromatase should be avoided. It is still speculative exactly what role aromatase plays in striatal neuron physiology. For the caudate-putamen, there is evidence that inhibition of aromatase prevents the induction of LTP in male rat MSNs (Tozzi et al., 2015) suggesting that local production of estradiol plays a role in striatal neuronal physiology. Inhibition of aromatase in the caudate-putamen of males proceeding a chemical lesion is neuroprotective (McArthur et al., 2007). To our knowledge, central administration of aromatase inhibitors has not been performed in females in studies examining striatal function.

Thus, the evidence for estradiol action in the striatal subregions is robust, but the source of that estradiol has not been directly tested in both sexes. One major question is the relationship between gonadal/peripheral vs. brain production of steroid sex hormones. The precursor to estradiol, testosterone, can increase the presence of aromatase expression and activity in rodent male brain (Roselli et al., 1984; Roselli and Klosterman, 1998), which is compelling evidence for the relationship of gonads and brain estradiol production in males. In male rats, long term testosterone exposure can influence MSN dendritic spine density (Wallin-Miller et al., 2016), and the nucleus accumbens is known to regulate the rewarding-aspects of testosterone exposure in males (Frye et al., 2002). It is unclear how castration and testosterone directly affect striatal aromatase activity and expression in males. For females, one study measuring estradiol content in both brain and blood of rodents across estrous stages found that estradiol content in the striatum was highest during late proestrus and far exceeded blood concentration (Morissette et al., 1992). However, at this point there remains a lack of corroborating evidence, especially when considered in light of the lack of differences in aromatase activity detected in other rat brain regions (Roselli et al., 1984). Continued research into how hormonal state and sex interact with possible aromatase activity is essential to grasp how steroid signaling modulates striatal neuron function.

## Author Contributions

AK wrote the initial manuscript draft. AK and JM revised and approved the manuscript.

### Conflict of Interest Statement

The authors declare that the research was conducted in the absence of any commercial or financial relationships that could be construed as a potential conflict of interest.
